# Numerical Investigation of Entropy Generation in Unsteady MHD Generalized Couette Flow with Variable Electrical Conductivity

**DOI:** 10.1155/2013/364695

**Published:** 2013-07-14

**Authors:** T. Chinyoka, O. D. Makinde

**Affiliations:** ^1^Center for Research in Computational and Applied Mechanics, University of Cape Town, Rondebosch 7701, South Africa; ^2^Institute for Advanced Research in Mathematical Modelling and Computations, Cape Peninsula University of Technology, P.O. Box 1906, Bellville 7535, South Africa

## Abstract

The thermodynamic second law analysis is utilized to investigate the inherent irreversibility in an unsteady hydromagnetic generalized Couette flow with variable electrical conductivity in the presence of induced electric field. Based on some simplified assumption, the model nonlinear governing equations are obtained and solved numerically using semidiscretization finite difference techniques. Effects of various thermophysical parameters on the fluid velocity, temperature, current density, skin friction, the Nusselt number, entropy generation number, and the Bejan number are presented graphically and discussed quantitatively.

## 1. Introduction

 Investigation of the flow of electrically conducting fluids in porous geometries is of particular importance due to the widespread prevalence in a number of industrial applications [1–4]. These include applications to geothermal reservoirs, nuclear reactor cooling, Magnetohydrodynamic (MHD) marine propulsion, electronic packaging, microelectronic device operations, thermal insulation, and petroleum reservoirs. Preliminary experimental evidence suggests promising future applications in the field of metallurgy, in particular in the MHD stirring of molten metals and in magnetic-levitation casting. Experimental and theoretical investigations of MHD flow in various geometries and under various conditions remain a topic of ongoing interest [5]. Similar investigations not including the effects of electrical conductivity are summarized in [6–12].

The cornerstone in the field of heat transfer and thermal design is the second law analysis and its design-related concept of entropy generation minimization. The foundation of knowledge of entropy production goes back to Clausius and Kelvin's studies on the irreversible aspects of the second law of thermodynamics. Since then, the theories based on these foundations have rapidly developed. However, the entropy production resulting from combined effects of velocity and temperature gradients has remained untreated by classical thermodynamics, which has motivated many researchers to conduct analyses of fundamental and applied engineering problems based on the second law analyses. Entropy generation is associated with thermodynamic irreversibility, which is common in all types of heat transfer processes, namely, conduction, convection, and radiation. In thermodynamical analysis, the fundamental principle remains the improvement of the relevant thermal systems to mitigate against energy losses and hence fully optimize the energy resources.

The purpose of this paper is to analyze the second law of thermodynamics with respect to inherent irreversibility in an unsteady flow of a viscous, incompressible, and electrically conducting fluid. The fluid flows through a channel with isothermal walls and a transversely imposed magnetic field. Expressions for the dimensionless velocity, temperature, current density, wall shear stress, all heat transfer, Bejan number, and entropy generation number are presented. These flow quantities are discussed qualitatively with respect to the embedded parameters. The mathematical formulation of the problem is established in Section 2. In Section 3, the semi-implicit finite difference technique is implemented for the solution process of the coupled nonlinear problem. Graphical results are presented and discussed qualitatively and quantitatively with respect to various parameters embedded in the system in Section 4.

## 2. Mathematical Model

**Figure 1:**
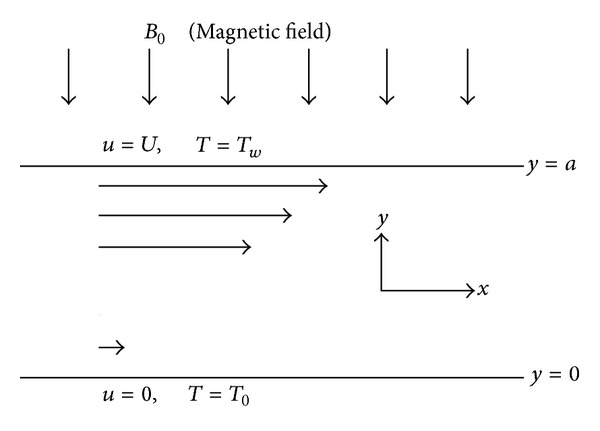
Geometry of the problem.

 The unsteady hydromagnetic generalized Couette flow of a viscous conducting incompressible fluid is considered in the presence of an imposed transverse magnetic field of strength *B*_0_ taking into account the induced electric field *E*_*z*_. The induced magnetic field is assumed to be small compared with the applied magnetic field and is neglected. Initially, at time $\overset{-}{t}\leq 0$, it is assumed that the fluid is stationary with temperature *T*_*i*_. At $\overset{-}{t}>0$, the fluid is subjected to a constant axial pressure gradient while the upper plate moves with a uniform velocity *U*. The plates' surface temperatures are non-uniform with temperature *T*_0_ at the lower plate and *T*_*w*_ at the upper moving plate such that *T*_*w*_ > *T*_0_ as shown in Figure 1.

The temperature-dependent variable electrical conductivity is given as [5]

(1) $$\begin{array}{c} \overset{-}{\sigma }=\sigma_{w}{\left(\frac{T-T_{0}}{T_{w}-T_{0}}\right)}^{\lambda }, \end{array}$$

where *σ*_*w*_ is the fluid electrical conductivity at the upper plate and *λ* is the electrical conductivity variation index. Under the above assumptions, the dimensionless governing equations for the momentum and energy balance can be expressed as

(2) Re∂w∂t=G−Ha(Le+w)θλ+∂2w∂η2,Re Pr∂θ∂t=∂2θ∂η2+Ec Pr(∂w∂η)2+Ec Pr Ha(Le+w)2θλ,

where *θ* is the dimensionless temperature, *w* is the dimensionless velocity, *t* is the dimensionless time, Re, Le, Ha, Ec, Pr, and *G* are the moving upper plate Reynolds number, the electric field loading parameter, magnetic field parameter, the Eckert number, the Prandtl number, and the pressure-gradient parameter. The appropriate initial and boundary conditions in dimensionless form are given as follows:

(3) $$\begin{array}{c} w\left(\eta ,0\right)=0,\;\;\theta \left(\eta ,0\right)=\theta_{i},\\ w\left(0,t\right)=0,\;\;\theta \left(0,t\right)=0, \end{array}$$

(4) $$\begin{array}{c} w\left(1,t\right)=0,\;\;\theta \left(1,t\right)=1. \end{array}$$

The following quantities have been utilized in order to obtain the dimensionless governing equations ((2) and (3)):

(5) η=ya,  t=Ut−a,  X=xa,  w=uU,θ=T−T0Tw−T0,  P=P−aμU,  θi=Ti−T0Tw−T0,  Re=ρhUμ,  Pr=μcpk,  G=−dPdX,Ec=U2cp(Tw−T0),  Le=EzUB0,  Ha=σwB02a2μ,

where *T* is the temperature, *U* is the uniform velocity of the upper plate, *a* is the channel width, *k* is the thermal conductivity, $\overset{-}{t}$ is the time, *μ* is the dynamic viscosity coefficient, *c*_*p*_ is the specific heat at constant pressure, and *P* is the fluid pressure. Other quantities of interest are the skin-friction coefficient (*C*_*f*_) and the Nusselt number (Nu) which are given as

(6) $$\begin{array}{c} C_{f}={\frac{\partial w}{\partial \eta }|}_{\eta =0,1},\;\;\text{Nu}={-\frac{\partial \theta }{\partial \eta }|}_{\eta =0,1}. \end{array}$$

The current density and the total current generated within the MHD flow system are given as

(7) $$\begin{array}{c} J_{z}=\theta^{\lambda }\left(\text{Le}+w\right)\;\left(\text{current}\;\text{density}\right),\\ I_{T}=\int_{0}^{1}\theta^{\lambda }\left(\text{Le}+w\right)d\eta \;\left(\text{total}\;\text{current}\right). \end{array}$$

It is important to note that, for a short circuit configuration where there is no electric field loading, Le = 0.

## 3. Entropy Analysis

 Hydromagnetic generalized Couette flow is inherently irreversible. This may be due to the exchange of energy between the conducting fluid and the moving plate surface. According to [13], the local volumetric rate of entropy generation for a viscous incompressible conducting fluid in the presence of magnetic field and induced electric field is given as

(8) $$\begin{array}{c} E_{G}=\frac{k}{T_{0}^{2}}\left(\frac{\partial T}{\partial \overset{-}{y}}\right)+\frac{\overset{-}{\mu }}{T_{0}}{\left(\frac{\partial \overset{-}{u}}{\partial \overset{-}{y}}\right)}^{2}+\frac{\overset{-}{\sigma }\left(E_{z}+uB_{0}\right)}{T_{0}}. \end{array}$$

The first term in (8) describes the heat transfer irreversibility while the second and third terms represent irreversibility due to fluid friction and magnetic field, respectively. Using (6), the dimensionless form of local entropy generation rate in (8) is given as

(9) Ns=a2T02EGk(Tw−T0)2=(∂θ∂η)2+BrΩ[(∂w∂η)2+Ha(Le+w)2θλ],

where *Ω* = (*T*_*w*_ − *T*_0_)/*T*_0_ is the temperature difference parameter and Br = Ec Pr is the Brinkman number. The Bejan number, Be, is defined as

(10) $$\begin{array}{c} \text{Be}=\frac{N_{1}}{N_{s}}=\frac{1}{1+\Phi }, \end{array}$$

where *N*_*s*_ = *N*_1_ + *N*_2_,

(11) $$\begin{array}{c} N_{1}={\left(\frac{\partial \theta }{\partial r}\right)}^{2},\\ N_{2}=\frac{\text{Br}}{\Omega }\left[{\left(\frac{\partial w}{\partial \eta }\right)}^{2}+\text{Ha}{\left(\text{Le}+w\right)}^{2}\theta^{\lambda }\right],\\ \Phi =\frac{N_{2}}{N_{1}}. \end{array}$$

*N*_1_ represents the irreversibility due to heat transfer, *N*_2_ represents fluid friction and magnetic field irreversibility, and Φ is the irreversibility ratio.

The Bejan number (Be) as shown in (10) has the range 0 ≤ Be ≤ 1. If Be = 0, then the irreversibility is dominated by the combined effects of fluid friction and magnetic fields, but if Be = 1, then the irreversibility due to heat transfer dominates the flow system by the virtue of finite temperature differences.

## 4. Numerical Solution

 Our numerical algorithm is based on semi-implicit finite difference schemes [14–19]. Implicit terms are taken at the intermediate time level (*N* + *ξ*), where 0 ≤ *ξ* ≤ 1. The discretization of the governing equations is based on a linear Cartesian mesh and uniform grid on which finite differences are taken. We approximate both the second and first spatial derivatives with second-order central differences. The equations corresponding to the first and last grid points are modified to incorporate the boundary conditions. The semi-implicit scheme for the velocity component reads:

(12) Re∂w∂t=G−Ha(Le+w(N+ξ))(θλ)(N)+∂2∂η2  w(N+ξ).

In (12), it is understood that ∂#/∂*t* : = (#^(*N*+1)^ − #^(*N*)^)/Δ*t*. The equation for *w* ^(*N*+1)^ then becomes

(13) −r1wj−1(N+1)+(Re+2r1+HaΔt[θλ](N))   ×wj(N+1)−r1wj+1(N+1)=explicit  terms,

where

(14) $$\begin{array}{c} r_{1}=\xi \frac{\Delta t}{\Delta \eta^{2}}. \end{array}$$

The solution procedure for *w* ^(*N*+1)^ thus reduces to inversion of tridiagonal matrices, which is an advantage over a fully implicit scheme. The semi-implicit integration scheme for the temperature equation is similar to that for the velocity component. Unmixed second partial derivatives of the temperature are treated implicitly as follows:

(15) Re Pr⁡∂θ∂t=∂2∂η2θ(N+ξ)+Ec Pr×[(∂w∂η)2+Ha(Le+w)2θλ](N).

The equation for *θ* ^(*N*+1)^ thus becomes

(16) −r1θj−1(N+1)+(Re Pr+2r1)θj(N+1)−r1θj+1(N+1)   =explicit  terms.

The solution procedure again reduces to inversion of tridiagonal matrices. The schemes ((13) and (16)) were checked for consistency. For *ξ* = 1, these are first order accurate in time but second order in space. The schemes in [14] have *ξ* = 1/2, which improves the accuracy in time to second order. As in [14–18] we, however, use *ξ* = 1 here so that we are free to choose larger time steps and still converge to the steady solutions.

## 5. Results and Discussion

 Unless otherwise stated, we employ the following parameter values:

*Re* = 1,  Pr = 0.71,  Ec = 1,  Ha = 0.2,  Le = 0.4,  *λ* = 0.1,  *θ*_*i*_ = 0, Δ*η* = 0.01,  Δ*t* = 0.01, *G* = 1, and  *t* = 10.

These will be the default values in this work. In the succeeding graphical, if any of these parameter values is not explicitly mentioned, it will be understood that such parameters take on the default values.

### 5.1. Transient and Steady-State Flow Profiles

**Figure 2:**
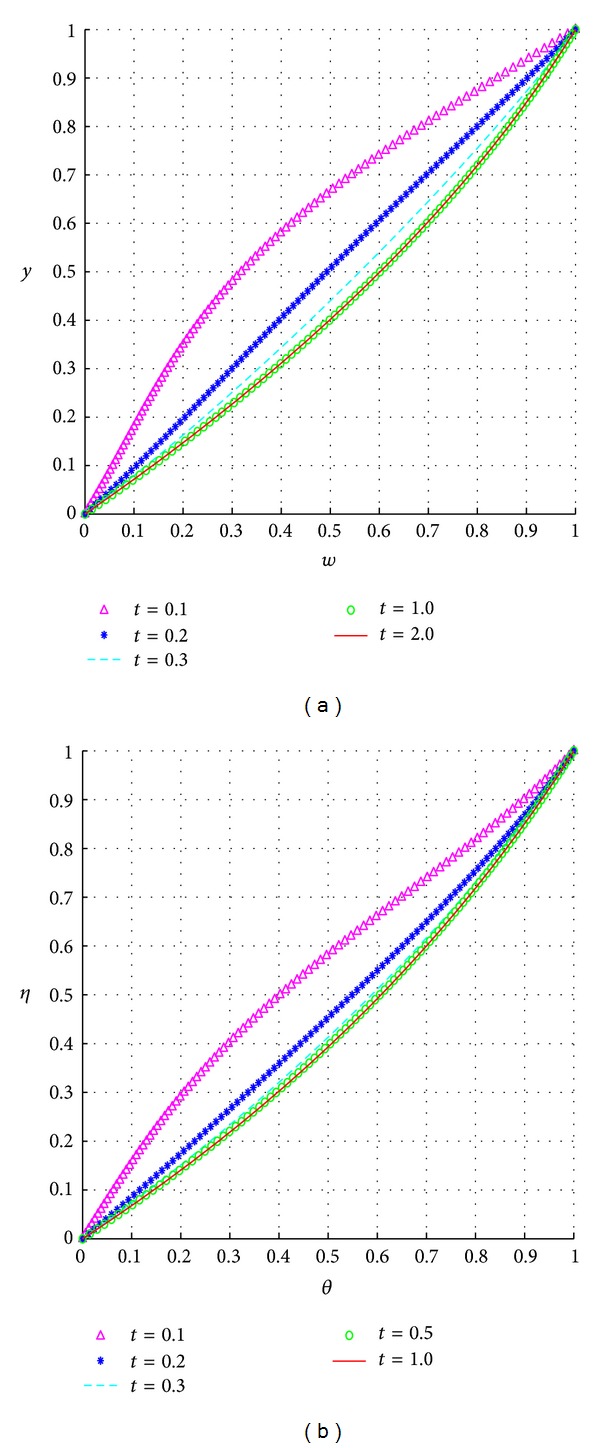
Transient and steady-state profiles.

 We display the transient solutions in Figure 2. The figures show a transient increase in both fluid velocity, Figure 2(a), and temperature, Figure 2(b), until a steady state is reached.

#### 5.1.1. Parameter Dependence of Solutions

**Figure 3:**
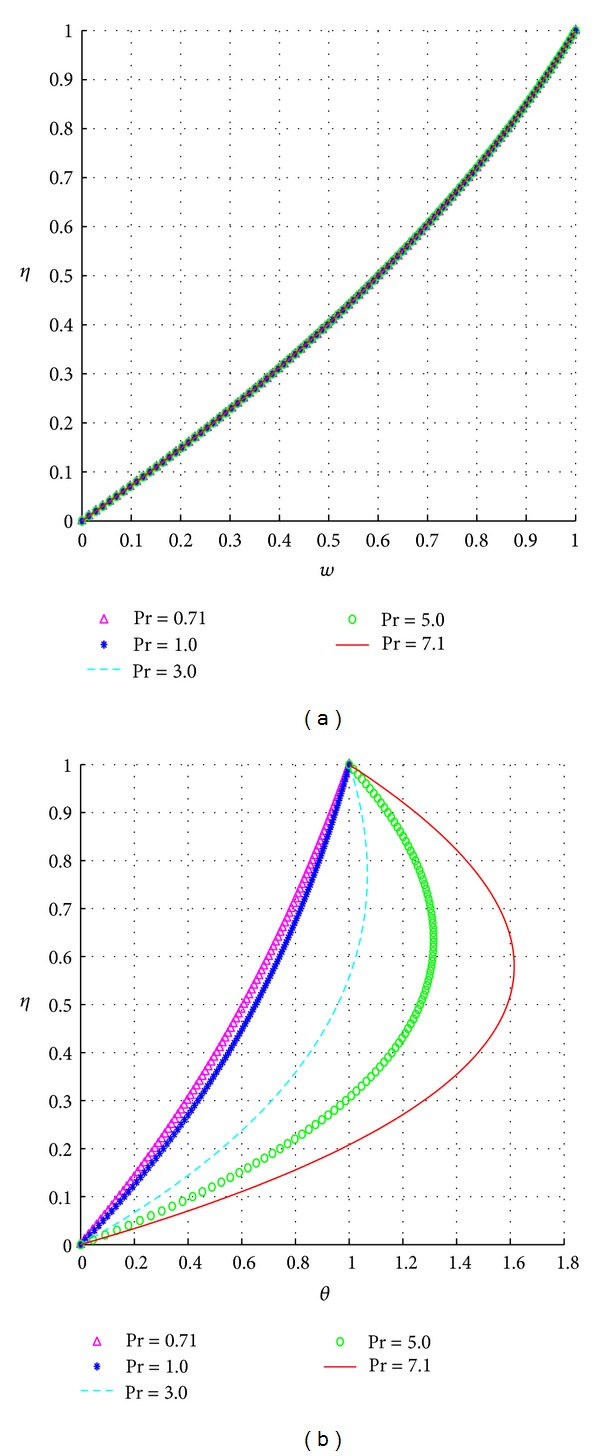
Effects of the Prandtl number, Pr.

 The response of the velocity and temperature to varying values of the Prandtl number (Pr) is illustrated in Figure 3. 

Larger values of the Prandtl number correspondingly increase the strength of the heat sources in the temperature equation, and hence this strength in turn increases the overall fluid temperature as clearly illustrated in Figure 3(b). Due to the implicit relationship between the Prandtl number and the velocity field via the magnetic field terms, we notice as expected that increases in the Prandtl number have limited effects on the fluid velocities as illustrated in Figure 3(a).

**Figure 4:**
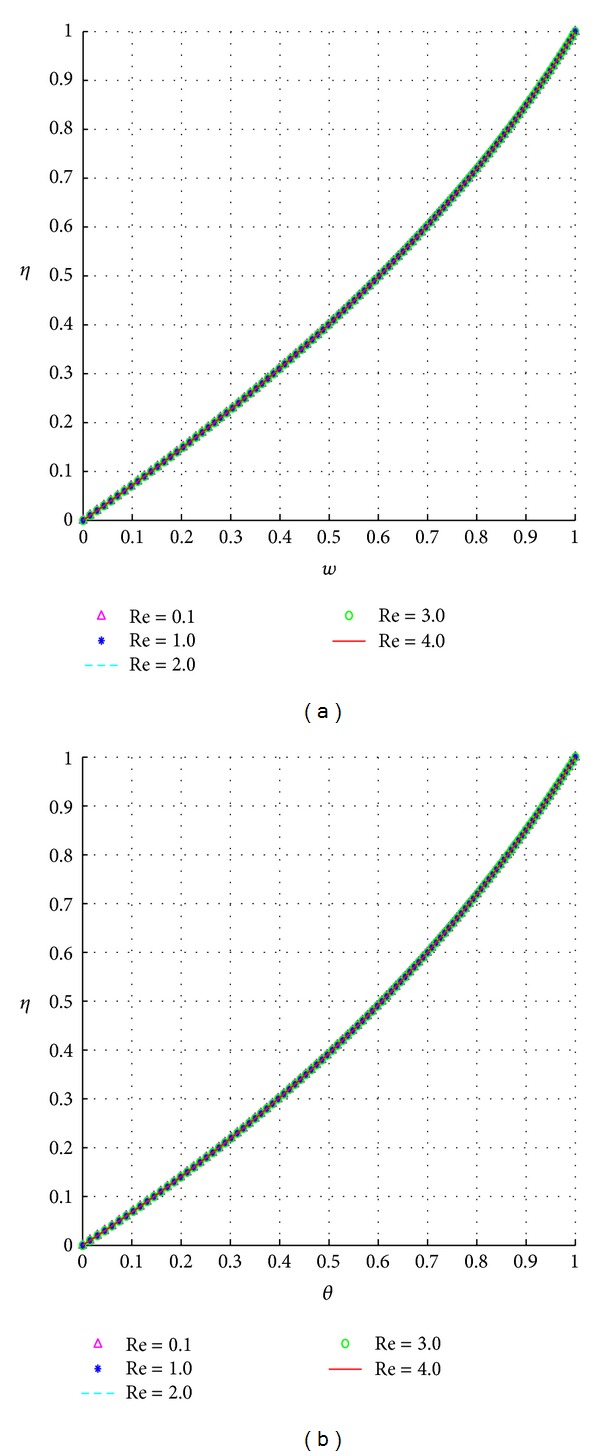
Effects of the Reynolds number, *Re*.

The response of the velocity and temperature to varying values of the Reynolds number (*Re*) is illustrated in Figure 4. 

The Reynolds number being coupled to the time-dependent terms shows limited effect on both the steady fluid velocity and temperature as shown in Figures 4(a) and 4(b).

**Figure 5:**
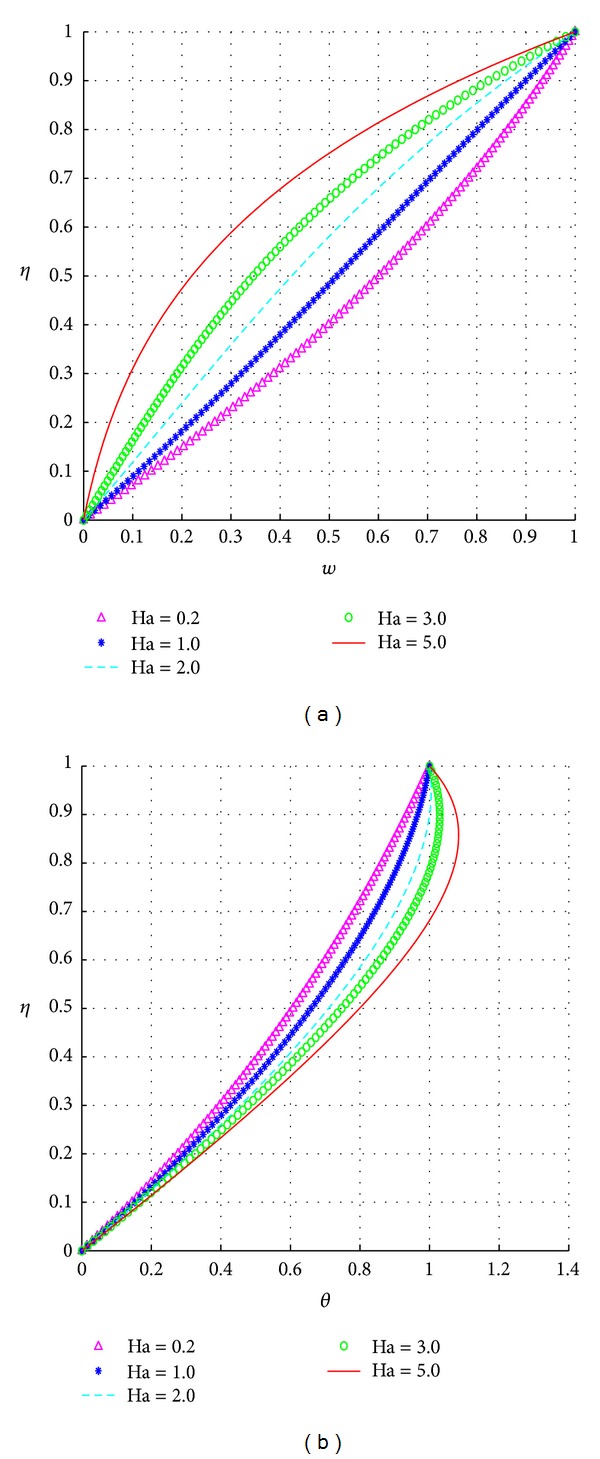
Effects of Ha.

The response of the velocity and temperature to varying values of the magnetic field parameter (Ha) is illustrated in Figure 5. 

Larger values of Ha correspondingly increase the strength of the heat sources in the temperature equation, and hence, this strength in turn increases the overall fluid temperature as clearly illustrated in Figure 5(b). On the other hand, higher values of Ha decrease the strength of the source terms in the velocity equation, and hence this decreased strength in turn increases the overall fluid temperature as clearly illustrated in Figure 5(a).

**Figure 6:**
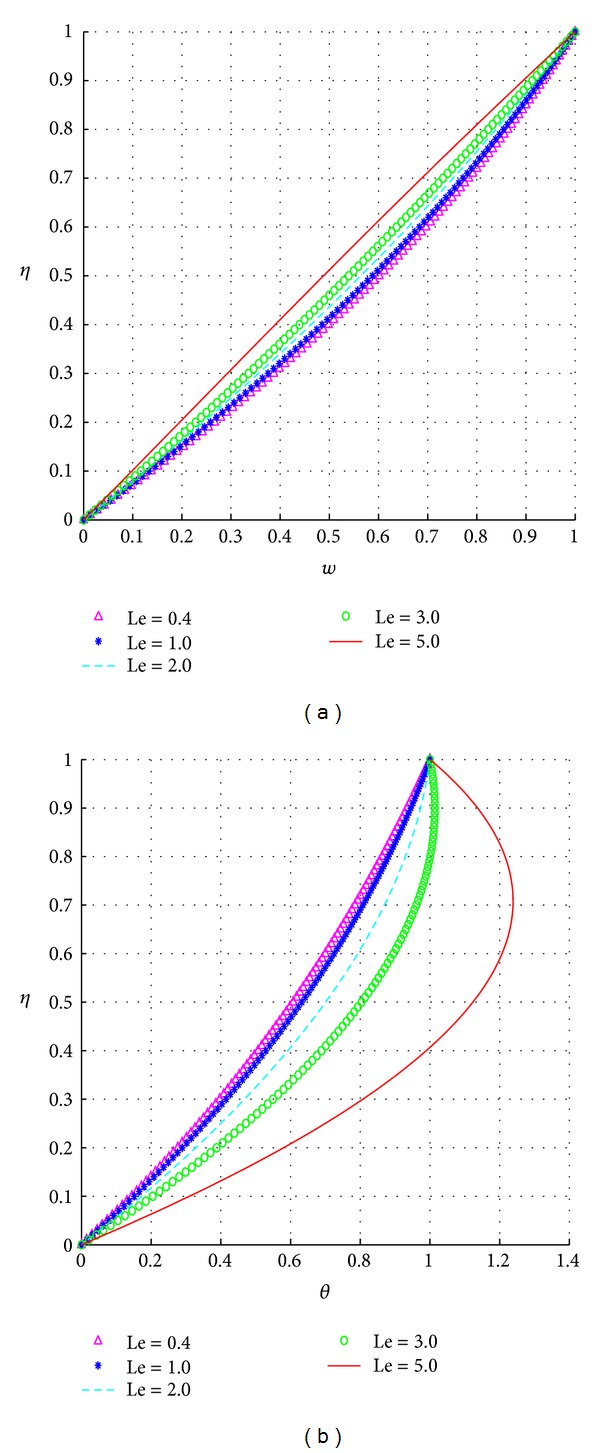
Effects of Le.

The effects of Le on both the velocity and temperature profiles are similar to those of Ha as illustrated in Figures 6(a) and 6(b). 

**Figure 7:**
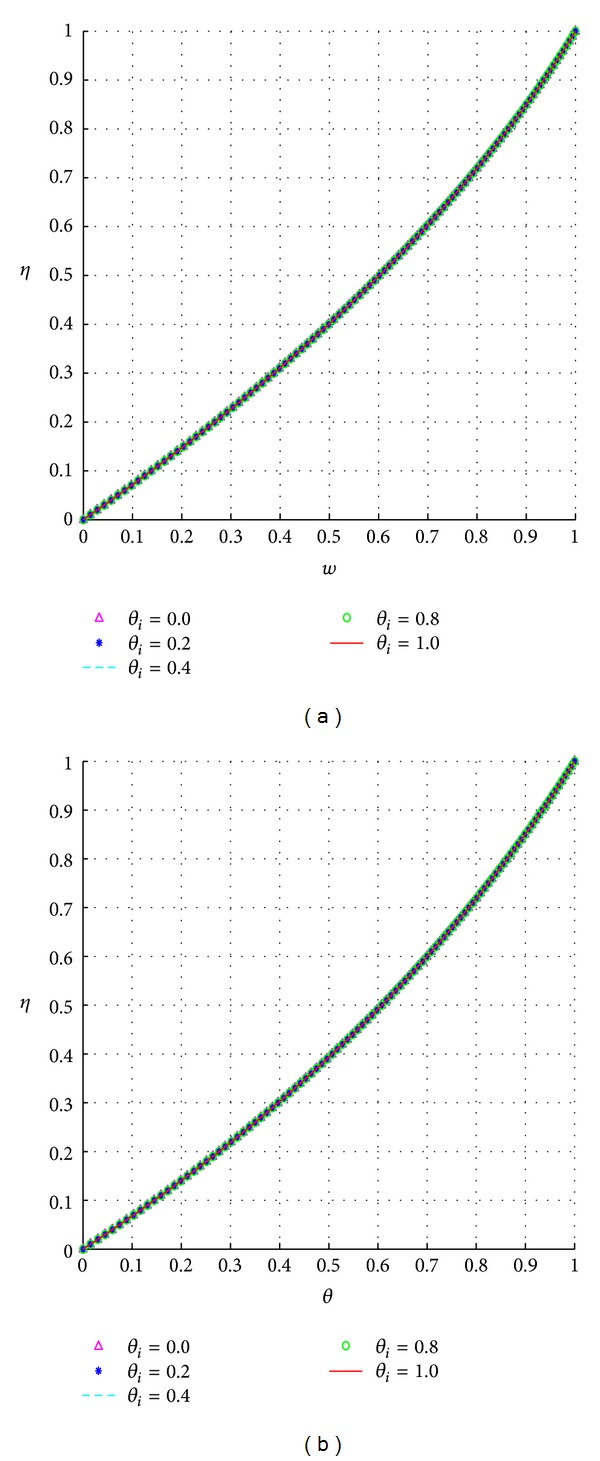
Effects of the initial fluid temperature, *θ*_*i*_.

Figures 7(a) and 7(b) show that the values of the initial temperature have limited effect on the steady-state flow profiles. 

**Figure 8:**
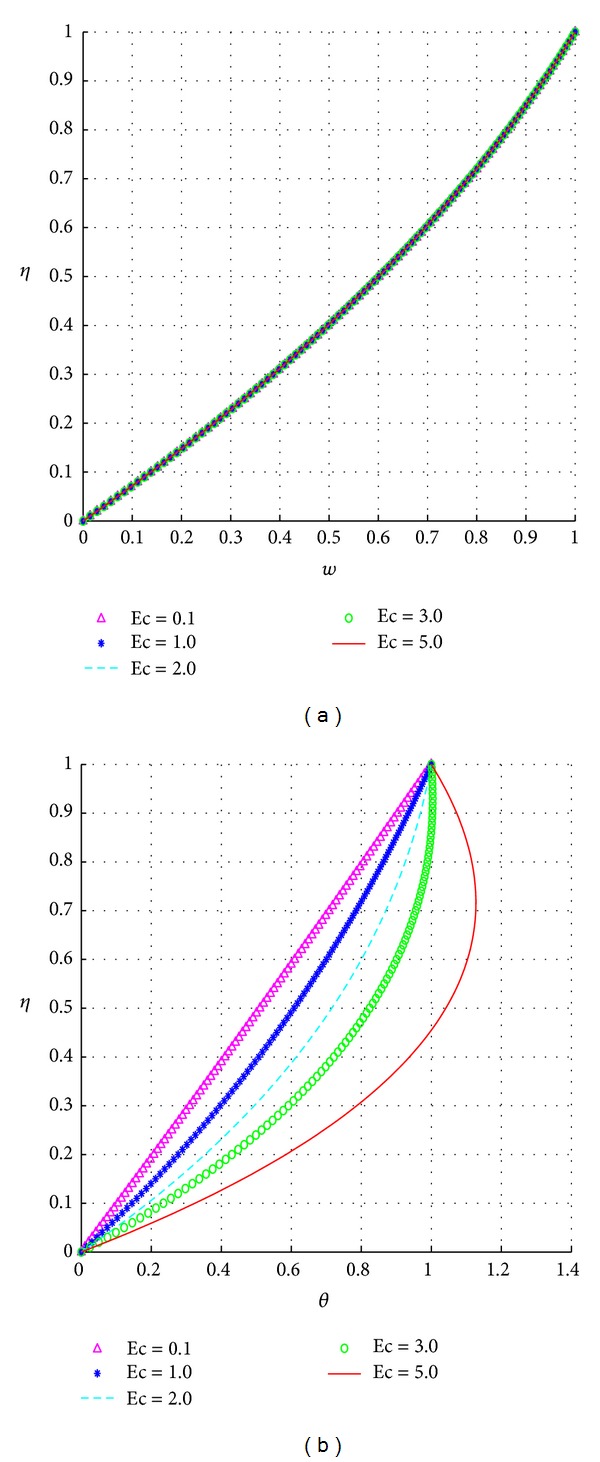
Effects of the Eckert number, Ec.

The response of the velocity and temperature to varying values of the Eckert number (Ec) are illustrated in Figure 8. 

**Figure 9:**
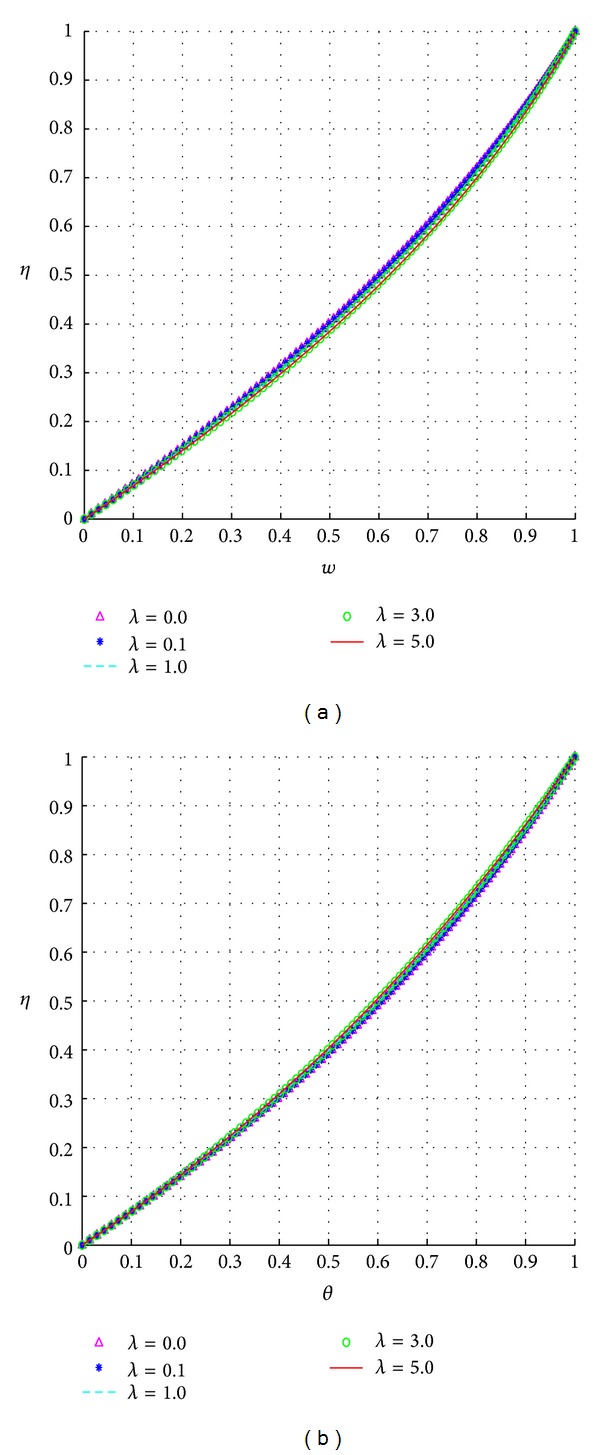
Effects of the electrical conductivity variation index, *λ*.

The effects of the Eckert number are similar to those for the Prandtl number. The effects of the electrical conductivity variation index on the velocity and temperature profiles are illustrated in Figure 9. 

As expected, since 0 ≤ *θ* ≤ 1, an increase in the electrical conductivity variation index (*λ*) correspondingly decreases the values of *θ* ^*λ*^. The source terms in the temperature equation will thus decrease in magnitude, whereas those in the velocity equation become less negative. This explains the slight decreases noticed in the fluid temperature, Figure 9(b), as well as the slight increases in the fluid velocity, Figure 9(a).

### 5.2. Wall Shear Stress and Wall Heat Transfer Rate

**Figure 10:**
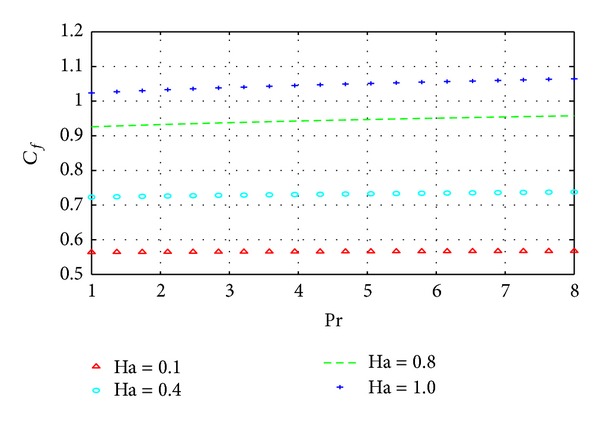
Variation of wall shear stress with Pr and Ha.

**Figure 11:**
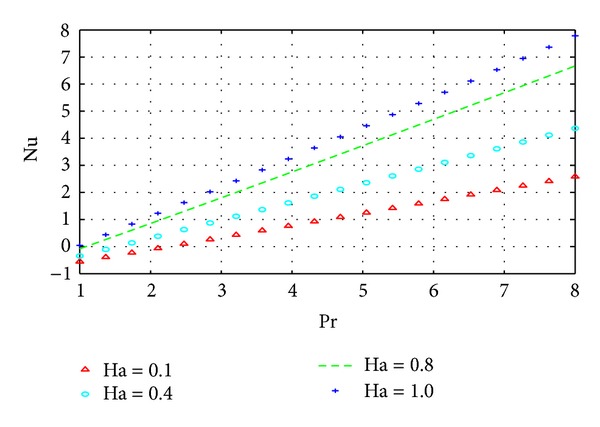
Variation of wall shear stress with Pr and Ha.

 The wall shear stress and wall heat transfer rates are illustrated in Figures 10 and 11, respectively, with varying Pr and Ha. 

**Figure 12:**
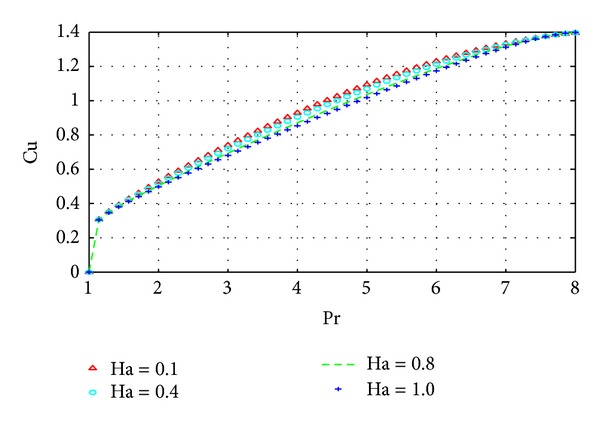
Variation of current density with Pr and Ha.

The results in Figures 10 and 11 simply summarize those in the previous section on parameter dependence of solutions. In particular, the behavior of the velocity and temperature with respect to certain parameters can be summarized in terms of the behavior of their respective gradients. The results of the current density similarly reflect the corresponding results on parameter dependence as shown in Figure 12.

### 5.3. Entropy Generation

**Figure 13:**
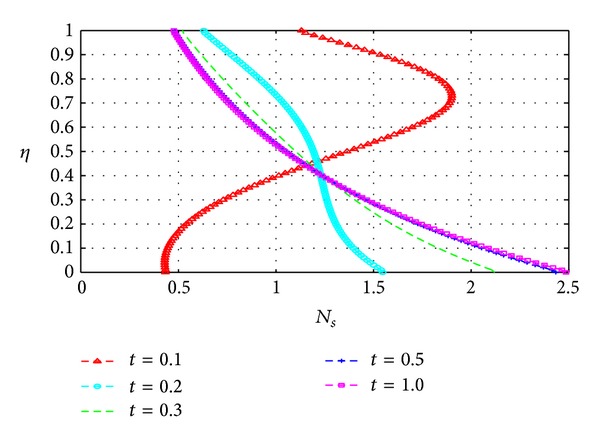
Variation of entropy generation rate with *η* and *t*.

 In this section, we plot the entropy generation rate (*N*_*s*_) across the channel under varying parameter conditions. Except for Figure 13, all graphs are otherwise drawn at the time *t* = 10. 

**Figure 14:**
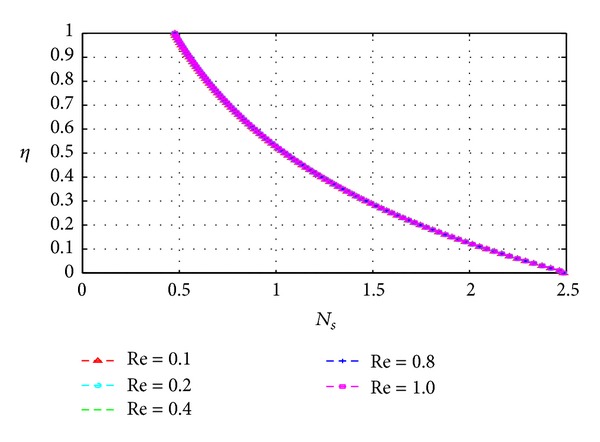
Variation of entropy generation rate with *η* and *Re*.

**Figure 15:**
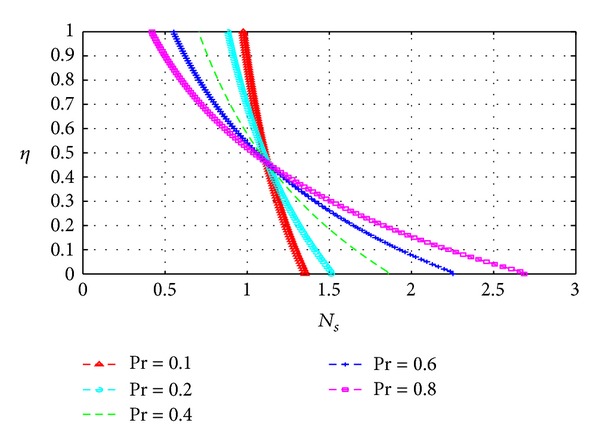
Variation of entropy generation rate with *η* and Pr.

**Figure 16:**
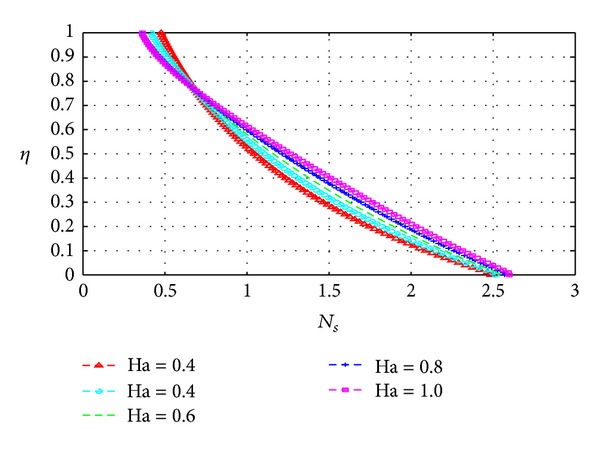
Variation of entropy generation rate with *η* and Ha.

**Figure 17:**
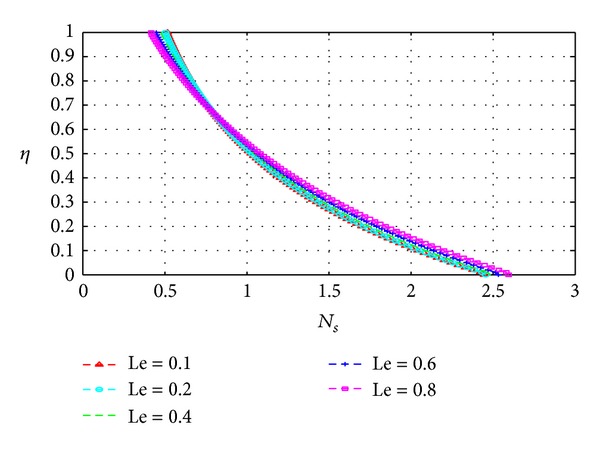
Variation of entropy generation rate with *η* and Le.

**Figure 18:**
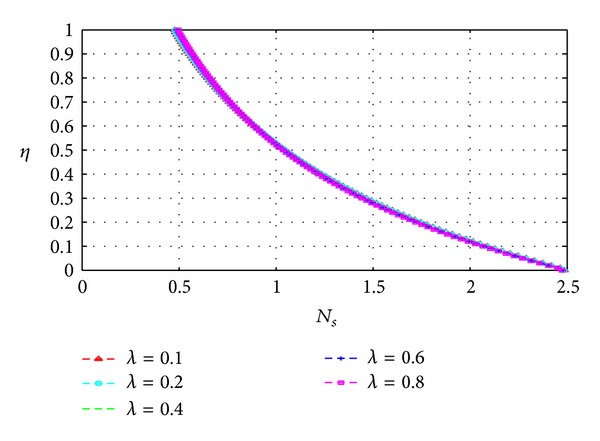
Variation of entropy generation rate with *η* and *λ*.

Figures 13, 14, 15, 16, 17, and 18 show the expected results for *N*_*s*_. In particular, parameters that increase the velocity and temperature gradients also increase the entropy generation rate and vice versa. In Figures 13–18, the values of the entropy generation rate expectedly vary across the channel including the walls in response to the evolution of the velocity and temperature gradients with increasing parameter values.

### 5.4. The Bejan Number

 In this section, we plot the Bejan number (Be) across the channel under varying parameter conditions. The analysis in this section is similar to that for the previous section with *N*_*s*_ now replaced by Be. 

**Figure 19:**
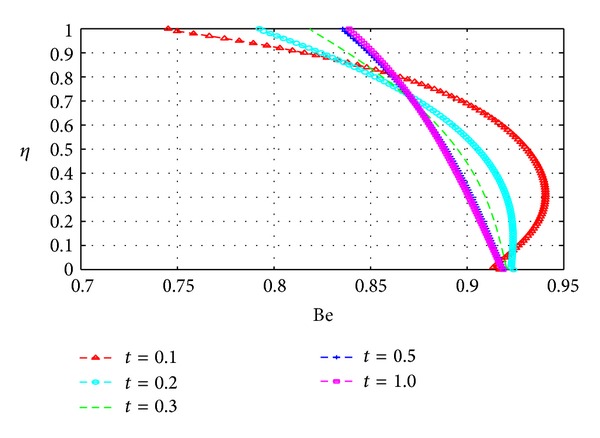
Variation of the Bejan number with *η* and *t*.

**Figure 20:**
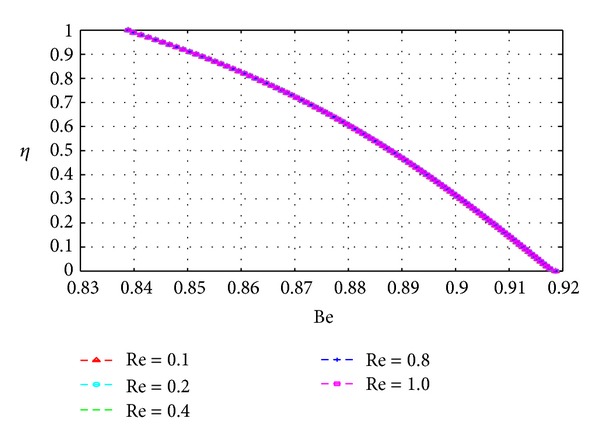
Variation of the Bejan number with *η* and *Re*.

**Figure 21:**
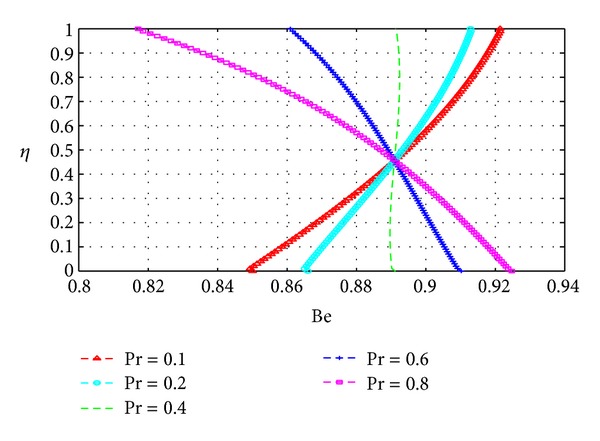
Variation of the Bejan number with *η* and Pr.

**Figure 22:**
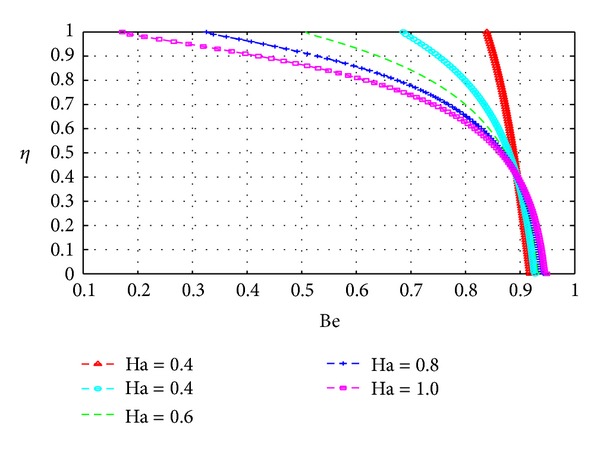
Variation of the Bejan number with *η* and Ha.

**Figure 23:**
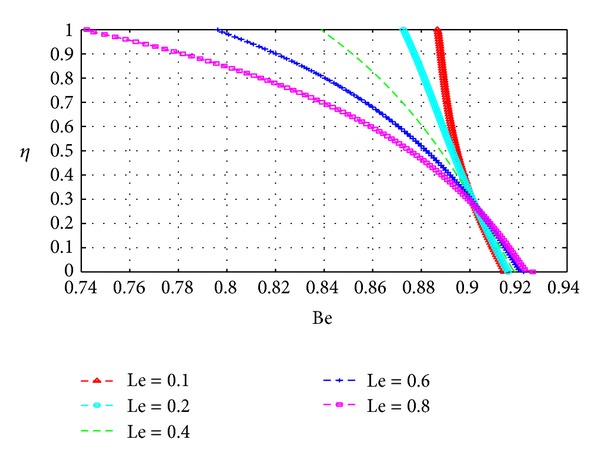
Variation of the Bejan number with *η* and Le.

**Figure 24:**
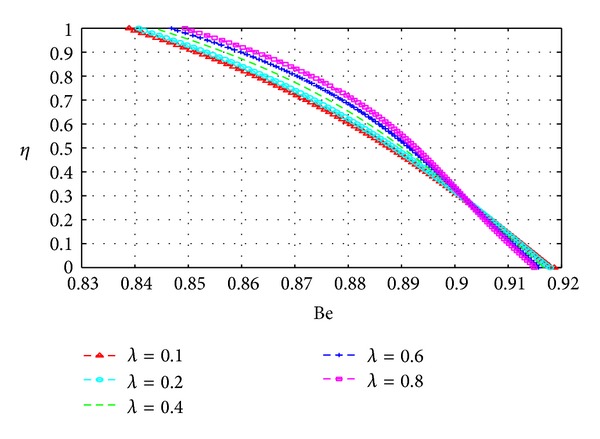
Variation of the Bejan number with *η* and *λ*.

Figures 19, 20, 21, 22, 23, and 24 show as expected that parameters which increase the entropy generation rate will correspondingly decrease the Bejan number and vice versa. In the vicinity of the walls, the strength of the fluid parameters will determine which mode of irreversibility dominates over the other.

## 6. Conclusion

 We computationally investigate the inherent irreversibility in an unsteady hydromagnetic generalized Couette flow with variable electrical conductivity in the presence of induced electric field. We also notice that, due to the nature of the source terms, the fluid velocity and temperature will each decrease (resp., increase) with a corresponding increase in the parameters that decrease/increase the magnitudes of the source terms. We have also demonstrated computationally that parameters which increase the entropy generation rate will correspondingly decrease the Bejan number and vice versa. In particular, as the flow profiles vary in shape from linear to “parabolic” in response to varying parameter values, the velocity/temperature gradients correspondingly change in magnitude leading to noticeable effects in the entropy generation rates and Bejan numbers.
